# Bio-Inspired Proprioceptive Touch of a Soft Finger with Inner-Finger Kinesthetic Perception

**DOI:** 10.3390/biomimetics8060501

**Published:** 2023-10-21

**Authors:** Xiaobo Liu, Xudong Han, Ning Guo, Fang Wan, Chaoyang Song

**Affiliations:** 1 Shenzhen Key Laboratory of Intelligent Robotics and Flexible Manufacturing Systems, Southern University of Science and Technology, Shenzhen 518055, China; 2 Department of Mechanical and Energy Engineering, Southern University of Science and Technology, Shenzhen 518055, China; 3 School of Design, Southern University of Science and Technology, Shenzhen 518055, China; 4 Guangdong Provincial Key Laboratory of Human-Augmentation and Rehabilitation Robotics in Universities, Southern University of Science and Technology, Shenzhen 518055, China

**Keywords:** object recognition, kinesthetic, humanoid finger, inner-finger vision, deep learning

## Abstract

In-hand object pose estimation is challenging for humans and robots due to occlusion caused by the hand and object. This paper proposes a soft finger that integrates inner vision with kinesthetic sensing to estimate object pose inspired by human fingers. The soft finger has a flexible skeleton and skin that adapts to different objects, and the skeleton deformations during interaction provide contact information obtained by the image from the inner camera. The proposed framework is an end-to-end method that uses raw images from soft fingers to estimate in-hand object pose. It consists of an encoder for kinesthetic information processing and an object pose and category estimator. The framework was tested on seven objects, achieving an impressive error of 2.02 mm and 11.34 degrees for pose error and 99.05% for classification.

## 1. Introduction

Humans exhibit various manipulative behaviors with the ability to detect the interaction behaviors of handled objects and hands. Visual information provides rich global features for humans to perceive objects’ shapes. However, without visual information, humans can still assess object properties, such as size, shape, position, and orientation, using the sense of touch alone [1]. There are many receptors in the skin at different depths on human hands to perceive mechanical stimuli during the interaction. Those receptors, cutaneous and kinesthetic, empower humans to feel objects relying on the sense of touch [2]. The cutaneous sense is the modality that depends on physical contact between skin and objects and is better for feeling the object’s pressure, vibration, and temperature. In contrast, the kinesthetic sense is the awareness of the position and movement of the body. It is better to feel the object’s position and orientation from the receptors within the muscles, tendons, and joints [3]. Inspired by the kinesthetic sense, we present a soft finger with an embedded camera and a deep learning architecture for object recognition.

According to the structure of human hands, many methods have been proposed for hand pose estimation (HPE) problems [4,5,6]. As the shape of an object and the configuration of a hand (how many fingers are used to manipulate objects and the fingers’ positions) are constrained by each other [7], some hand–object joint detection methods are proposed, which are called hand–object pose estimation (HOPE) [8,9,10]. Like humans manipulating objects with HOPE, object pose recognition is also a fundamental and challenging task in robotics.

For a manipulation task, perception of the environment and objects is essential [11]. Vision sensors are standard solutions to perceive the environment, and many methods have been proposed for object localization and classification [12,13,14,15]. While deep learning significantly improves performance in object recognition problems, the inevitable occlusion is still challenging, especially in dexterous manipulation tasks. Even if we obtain an object pose with high precision before manipulation, the pose during manipulation in the gripper is still unknown due to the inherent uncertainties, tolerances, and noise in the robotic system [16].

Inspired by the HOPE problem, we try to solve the robot gripper–object pose estimation problem with gripper pose estimation. For fully actuated grippers, we can obtain the joints’ angles of the gripper from motors and contact states from tactile and force sensors and estimate object pose and category with that information [17,18,19]. For under-actuated grippers, we need additional sensors to measure extra degrees of freedom (DoF), then estimate object pose and category [20,21,22]. To measure the contact information, GelSight [23], DIGIT [24], and FingerVision [25] are proposed. Those sensors consist of a transparent hard layer and a thin elastic layer and predict object shape and contact force from the deformation of the elastic layer. Limited by the thickness of the elastic layer, which is 2–3 mm, the sensors’ deformation is small, limiting the measuring range and shape adaptation during grasping.

**Figure 1:**
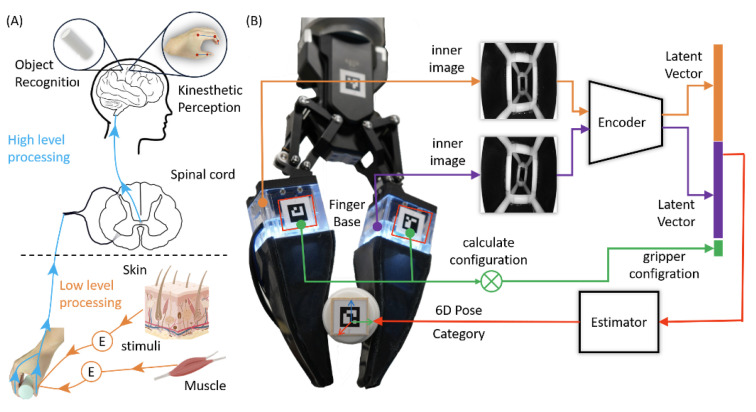
Overview of the bio-inspired finger and framework. (**A**) The raw stimulus is encoded with low-level processing and then transmitted to the central nervous system (CNS) for high-level processing, such as object recognition. (**B**) The bio-inspired fingers with flexible skeleton and silicon gel skin and a framework for object recognition: the raw images from fingers are encoded as latent vectors and then used for auxiliary tasks such as pose estimation and object classification.

Those methods mentioned above use multi-sensors in fingers for joints and tactile sensors in the fingertip for contact states and then estimate the objects’ poses and categories with CAD models. In this article, we propose a soft, adaptive finger with an integrated camera to infer the finger deformation during interaction with objects, as shown in Figure 1. We mount the soft fingers on a gripper to enhance the adaption of the gripper and recognize handle objects with their proprioceptive sensing. Our method uses raw images to estimate objects’ poses and categories with a camera and unknown CAD models. Instead of a two-stage method to recognize gripper state and object pose, our method is one-stage to recognize handled objects’ poses and categories from the raw images. To simplify the training and enhance the reusability, we split the method into two parts: a feature extractor for interaction information embedding and a post-processor for further manipulation tasks. The feature extractor is an encoder–decoder architecture with ResNet block [26], and the post-processor is a multilayer perceptron (MLP) for classification and regression. The main contributions of this paper include the following: First, we design and fabricate a soft finger with an integrated camera inside for proprioception. Second, we propose a frame to extract and fuse the fingers’ data for objects’ states in a gripper. Finally, we test the effectiveness of the proposed method and obtain high accuracy in pose estimation and classification.

## 2. Materials and Methods

### 2.1. Design and Fabrication of the Soft Finger with Inner Vision

**Figure 2:**
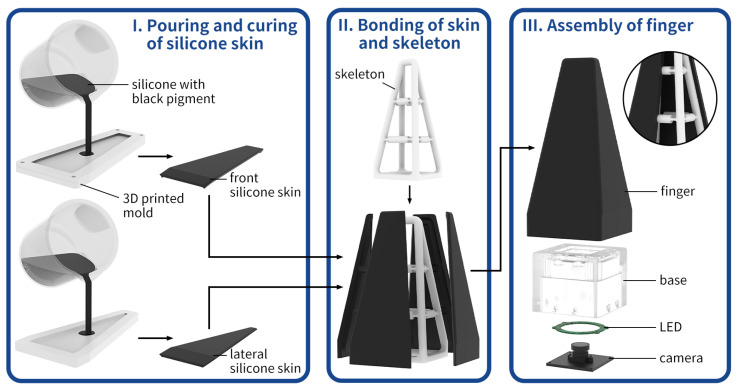
The soft finger’s design and fabrication. (**I**) The fabrication process of the silicone skin; (**II**) attaching the skin to the basic finger skeleton; (**III**) the integrated finger with an LED and an inner camera.

In our previous work [27], we leveraged the soft finger with an AruCo marker inside to sense contact force and torque, which encodes the deformation of the finger. In this study, we introduce several improvements to the finger design, as shown in Figure 2:

- Added silicone skin on the finger to isolate the outside environment for a clear background.
- Added an LED light for illumination as the skin blocked the outside light.
- Removed the AruCo marker and used the finger’s skeleton as a deformation feature.

As shown in Figure 2, this new design finger contains a finger skeleton with black skin, a base frame, an LED light, and a camera. The finger skeleton was fabricated with vacuum molding using polyurethane elastomers (Hei-cast 8400 from H&K). The three components were mixed in a 1:1:0 proportion to achieve 90A hardness with robust performance according to previous experience. Alternatively, other fabrication methods, such as fused deposition modeling (FDM) or stereolithography (SLA), could be cheaper. The size of the skeleton is 50 mm in bottom side length and 120 mm in height. The skin is made of Smooth-On Ecoflex${}^{\mathrm{TM}}$ 00-30 silicone rubber, and we added black pigment to change the color to block the ambient light effectively. Moreover, the silicone skin’s thickness is 3 mm, fabricated individually, and attached to the finger skeleton with an adhesive Valigooo${}^{®}$ V-80. The white LED light has enough luminous flux for the camera’s exposure. The chosen camera is Chengyue WX605 from Weixinshijie, with a 640 × 360 resolution at 330 fps, and the lens is manually adjustable. When grasping objects, the deformed finger could be captured by the camera and encoded as the interaction information. Therefore, we use this proposed finger to recognize the outside object.

### 2.2. Framework for Handled Object Recognition with the Soft Finger

**Figure 3:**
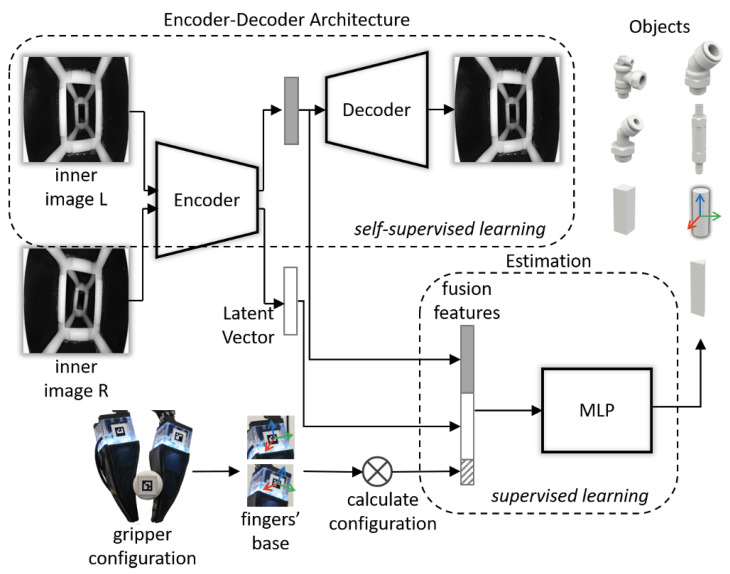
The architecture of the proposed framework: it takes two resized grayscale images and a gripper configuration as inputs and predicts the 6D pose and category of the object.

In this section, we present a framework illustrated in Figure 3 to extract kinesthesia features and estimate the object state handled by the gripper. This framework contains two parts: an encoder–decoder architecture for feature extraction and two auxiliary multilayer perceptrons (MLP) for estimating the object’s pose relative to the gripper’s coordinate system and category, respectively. The input of the framework is two fingers’ inner images and the gripper configuration.

#### 2.2.1. Encoder–Decoder Architecture

**Figure 4:**
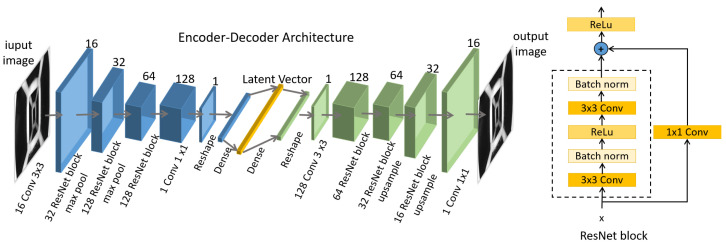
The encoder–decoder architecture of the feature extraction: one resized grayscale image as inputs and the same size image as output.

Specific details of the encoder–decoder architecture are shown in Figure 4; the blue block is the encoder, the green block is the decoder, and the yellow vector is the extracted latent vector. The encoder–decoder architecture is a fully convolutional topology and takes a resized grayscale image $I=R^{1\;\times \;320\;\times \;320}$ as input. It extracts the features representing the finger’s deformation and outputs an N-dimensional vector; the decoder reconstructs the image from the feature vector.

The basic blocks of the encoder–decoder architecture are 3 × 3 convolution and ResNet block for extracting features and 1 × 1 convolution for compressing features. The dense layer is set to change the latent vector’s dimensions and explore the feature dimensions’ effect on recovering the image.

Define the input as *I*, the encoder function as *E*, the latent vector as *V*, the decoder function as *D*, and the output as *Z*. The encoder–decoder architecture can be described as

(1) $$\begin{array}{c} V=E(I), \end{array}$$

(2) $$\begin{array}{c} Z=D(V), \end{array}$$

(3) $$\begin{array}{c} \left({\hat{\theta }}_{e},{\hat{\theta }}_{d}\right)=\underset{\theta_{e},\theta_{d}\in \Theta }{arg}\min Loss\left(Z,I\right) \end{array}$$

where ${\hat{\theta }}_{e},{\hat{\theta }}_{d}$ are the well-trained encoder and decoder parameters, and Loss is the loss function between *Z* and *I*.

#### 2.2.2. Pose Estimation and Classification

**Figure 5:**
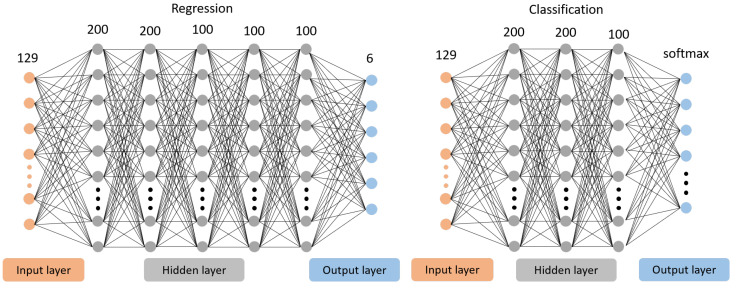
Two MLP models of object recognition. Left: a regression model for 6d pose estimation. Right: a classification model for object categories.

After extracting the latent feature *V*, we designed two MLP models to estimate the object’s pose and category as shown in Figure 5. These two models have the same inputs, and the output of the regression model is a 6D pose. In contrast, the output of the classification model is seven classes with a softmax activation function. In this article, the input vector is 129 dimensions, aggregating the two fingers’ feature *V* and gripper configuration. In the follow-up work, we set the dimension of *V* as 64 and the dimension of the gripper configuration as one since the gripper we used is one degree of freedom (DoF), so the input vector is 64×2+1=129 dimensions. The regression model consists of five hidden layers with 200, 200, 100, 100, and 100 neurons, with an activation function rectified linear unit (ReLu) [28] and batch normalization [29]. The classification model consists of three hidden layers with 200, 200, and 100 neurons, with activation function ReLu.

Define two images taken from the inner cameras of the fingers as $I^{L}=R^{C\;\times \;H\;\times \;W}$, $I^{R}=R^{C\;\times \;H\;\times \;W}$ with height *H* and width *W*, gripper configuration as $G_{c}$, regression model as $F_{r}$, classification model as $F_{c}$, object 6d pose as $S_{p}$, and object category as $S_{c}$, and the two MLP models are described as

(4) $$\begin{array}{c} V_{L},V_{R}=E\left(I_{L}\right),E\left(I_{R}\right), \end{array}$$

(5) $$\begin{array}{c} V_{aggregation}=Func\left(V_{L},V_{R},G_{c}\right), \end{array}$$

(6) $$\begin{array}{c} S_{p}=F_{r}\left(V_{aggregation}\right),S_{c}=F_{c}\left(V_{aggregation}\right), \end{array}$$

Here, Func is a function to combine the vectors in order.

### 2.3. Data Collection and Training Setups

#### 2.3.1. Data Collection Setup

**Figure 6:**
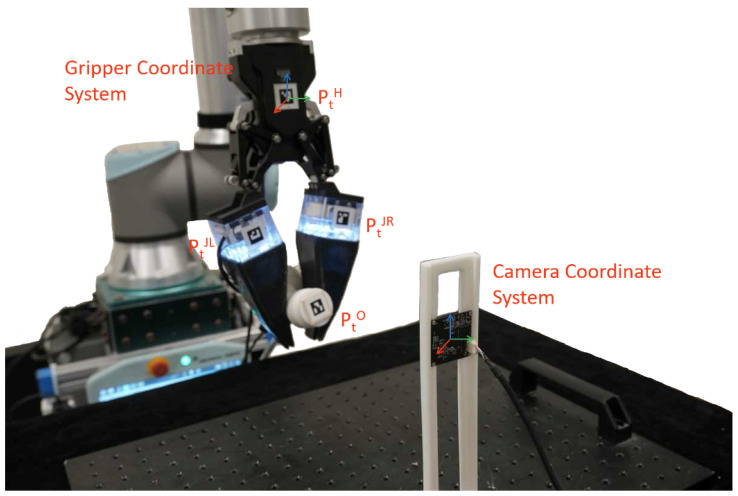
Date collection setup. Four markers are attached to the gripper, fingers, and object. An outside camera monitors the four markers for the object’s pose; simultaneously, fingers’ deformations are captured by two inner cameras.

We built an experimental platform to collect training data efficiently for training the framework above, as shown in Figure 6. The designed fingers are mounted on a DH-Robotics AG-160-95 adaptive gripper to replace its tips, and we pasted AruCo codes on the fingers and grippers to represent their poses. An extra camera is mounted on an optical breadboard to collect the AruCo marker poses, and two cameras in the fingers collect the interaction deformations. The AruCo markers are 4 × 4 squares of 16mm width with different indexes and are detected by OpenCV [30]. The outside camera’s resolution is set at 1920 × 1080 to increase the detection success rate and precision of AruCo markers detection. The inner camera’s resolution is 640 × 360 and resized to 320 × 320 to decrease the model’s size and prediction time.

**Figure 7:**
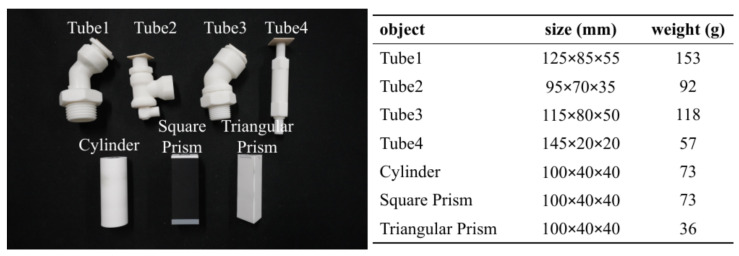
Test objects and their properties. (**Left**): 3D-printed objects. (**Right**): objects’ sizes and weights.

Referring to article [31], we chose the McMaster dataset (https://www.mcmaster.com accessed on 20 June 2023) as our test objects. In addition, we also chose three basic geometric solids. All objects are resized to adjust the gripper width and 3D-printed for final usage as shown in Figure 7. When collecting data, we set the gripper force mode and control the gripper width to grasp the object, then leave the gripper static and shake the object manually to collect the object poses. Due to the finger’s adaptation, the gripper width does not need to precisely match the objects’ size, which is predefined at 15 mm. We collected 5000 samples for each object.

After collection, all poses are transferred to the gripper coordinate system for standardization. Define P=(x,y,z,rx,ry,rz) as a pose, where (x,y,z) is translation and (rx,ry,rz) is orientation. Instead of using the object CAD model, we use the relative change to represent the object’s pose without the object model. Define reference pose $P_{0}=\left(x_{0},y_{0},z_{0},rx_{0},ry_{0},rz_{0}\right)$, current pose $P_{t}=\left(x_{t},y_{t},z_{t},rx_{t},ry_{t},rz_{t}\right)$ at time *t*, and the translation matrix $M_{t}=\left[R|T\right]$, so

(7) $$P_{t}=P_{0}M_{t},$$

and we use $M_{t}$ to represent the current object pose.

The left superscript *G* and *C* represent the gripper and camera coordinate system variables. *G* is the gripper coordinate system, and *C* is the camera coordinate. The in-camera coordinate system, the gripper pose, gripper configuration, and object pose indicated by the Aruco marker attached are ${}^{C}P_{t}^{O}$, gripper poses ${}^{C}P_{t}^{H}$, gripper joint poses ${}^{C}P_{t}^{JL}$ and ${}^{C}P_{t}^{JR}$ in time *t*.

The transfer to gripper coordinate system is as follows:

(8) $$\begin{array}{c} {}^{C}P_{t}^{O}={}^{C}P_{t}^{H}\cdot {}^{G}P_{t}^{O}, \end{array}$$

(9) $$\begin{array}{c} {}^{G}P_{t}^{O}={{[}^{C}P_{t}^{H}]}^{-1}\cdot {}^{C}P_{t}^{O}, \end{array}$$

(10) $$\begin{array}{c} {}^{G}P_{0}^{O}={{[}^{C}P_{0}^{H}]}^{-1}\cdot {}^{C}P_{0}^{O}, \end{array}$$

(11) $$\begin{array}{c} {}^{G}P_{t}^{O}={}^{G}P_{0}^{O}\cdot^{G}M_{t}, \end{array}$$

In the gripper coordinate system, the object transfer pose ${}^{G}M_{t}$ is

(12) $$\begin{array}{cc} {}^{G}M_{t} & ={{[}^{G}P_{0}^{O}]}^{-1}\cdot {}^{G}P_{t}^{O}\\ \; & ={\left[{{[}^{C}P_{0}^{H}]}^{-1}\cdot {}^{C}P_{0}^{O}\right]}^{-1}\left[{{[}^{C}P_{t}^{H}]}^{-1}\cdot {}^{C}P_{t}^{O}\right] \end{array}$$

**Figure 8:**
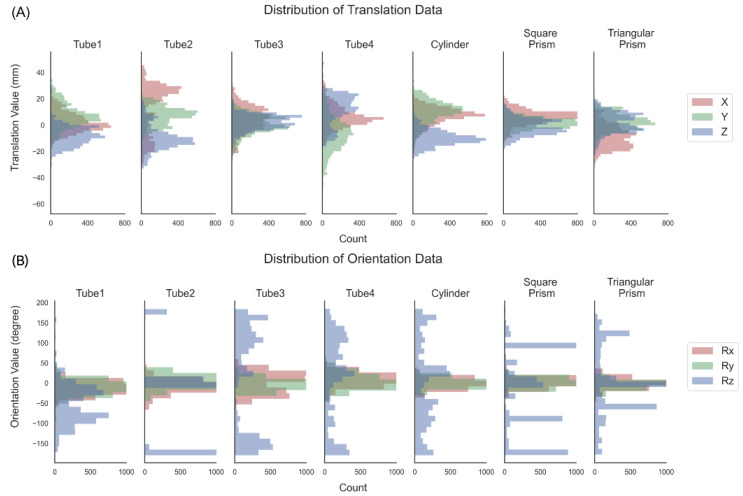
Distribution of the dataset. All data are exhibited in the gripper coordinate system: (**A**) translation distribution and (**B**) orientation distribution.

The collected dataset comprises seven objects and 5000 samples per object, each consisting of two inner images, four poses from the outside camera, and the objects’ categories. The resolution of the inner images is the same and is 640 × 360, and resized to 320 × 320 for input, and the values are normalized to 0–1. The objects’ pose distributions are shown in Figure 8.

#### 2.3.2. Network Training Setup

To improve the reusability and expansibility of the network, we trained the encoder–decoder reconstruction and the auxiliary tasks in two stages using the dataset collected in the previous section.

In the first stage, the encoder–decoder reconstruction is self-supervised learning. The dataset is randomly split into 8:2; 56,000 images are used for training, and 14,000 are used for evaluation. It was trained with a batch size of 32 using an Adam optimizer with a learning rate of 0.001 on mean squared error loss (MSELoss). The latent vector *V* is set to 8, 16, 32, 64, 128, and 256 to determine the best network configuration. The training epoch is set to 200, and we save the weights with the lowest training loss.

We froze the encoder’s weights in the second stage and only trained the following auxiliary tasks. For the regression model, we trained an MLP model for each object. Using the split dataset above, 4000 samples are used for training, and 1000 are used for evaluation for each object. The batch size is 32, the optimizer is Adam optimizer, and the learning rate is 0.001. As the 6D pose consists of two parts, translation, and orientation, and we define the training loss in Equations (13)–(15), where $L_{t}$ is translation loss and $L_{r}$ is orientation loss. The hyper-parameters α and β are set to 0.01 and 10, while the translation unit is millimeters and rotation is radians. The training epoch is set to 100.

(13) $$\begin{array}{c} L_{t}=\frac{1}{3}\sum_{n=1}^{3}{\left(x_{n}^{t}-{\hat{x}}_{n}^{t}\right)}^{2}, \end{array}$$

(14) $$\begin{array}{c} L_{r}=\frac{1}{3}\sum_{n=1}^{3}{\left(x_{n}^{r}-{\hat{x}}_{n}^{r}\right)}^{2}, \end{array}$$

(15) $$\begin{array}{c} L=\alpha L_{t}+\beta L_{r}. \end{array}$$

For the classification model, we trained an MLP model for all objects together. Using the split dataset above, 28,000 samples are used for training and 7000 for evaluation. The batch size is 256, the optimizer is the Adam optimizer, the learning rate is 0.001, and the training loss is cross-entropy loss. The training epoch is set to 100.

## 3. Results and Discussion

### 3.1. Dimension of the Latent Vector

To find an optimal dimension of the latent space, we varied the dimension of the latent vector and compared the reconstruction errors using the same training and validation dataset. The dimension of the latent vector is set to 8, 16, 32, 64, 128, and 256, and the corresponding results are shown in Table 1.

We scaled all losses such that the loss of 256-dimensional latent space was one. As the dimension increases, the reconstruction loss decreases, and the number of parameters of the model increases. To balance the precision and computational efficiency of the auto-encoder, we chose the 64-dimensional latent space, whose loss is comparable to the 256-dimensional space with only 24% of the number of parameters.

### 3.2. Quantitative Evaluation of Object Recognition

In this section, we report the accuracy of pose estimation and classification. The translation error is measured by the Euclidean distance $∥p_{est}-p_{gt}{∥}_{2}$ between the estimated position $p_{est}={\left(x,y,z\right)}_{est}$ and the ground truth position $p_{gt}={\left(x,y,z\right)}_{gt}$ [32]. The orientation error |α|, measured by an absolute angle error, is computed as

(16) $$\begin{array}{c} 2\cos|\alpha |=\mathrm{Tr}(R_{gt}^{-1}R_{est})-1, \end{array}$$

where $R_{gt}$ and $R_{est}$ are the estimated and ground truth rotation matrices, and Tr is the trace of the matrix.

**Figure 9:**
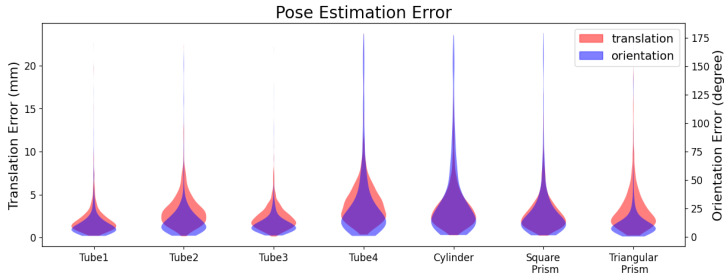
The histogram of pose estimation errors of each object. Translation error is the Euclidean distance, and rotation error is the absolute orientation error |α|.

**Figure A1:**
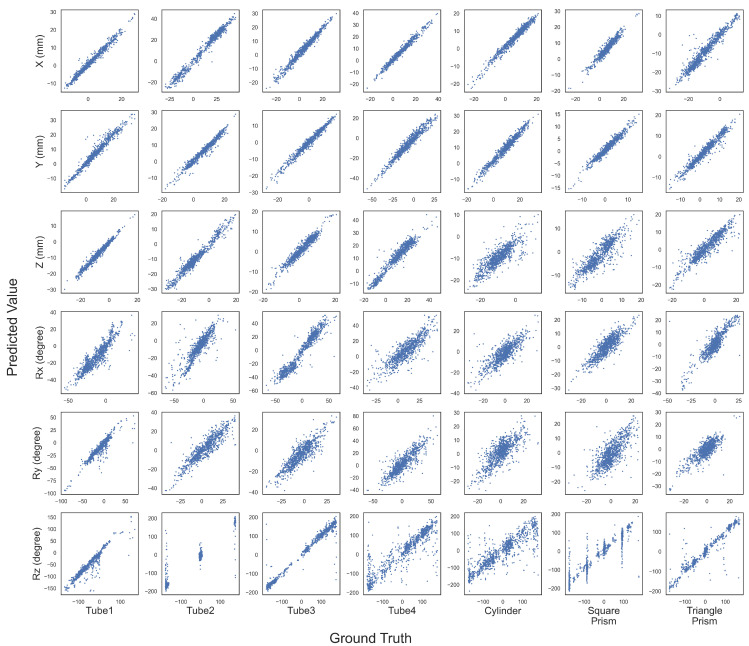
Results of the pose estimation for test objects. Six rows correspond to positions and orientations, and seven columns correspond to seven objects. The x-axis is the ground truth and the y-axis is the predicted values.

As shown in Figure 9, the translation and orientation errors are significantly different for different objects. The mean translation error of each object is between 2.02 mm and 4.00 mm, and the mean orientation error is between 11.34 degrees and 31.87 degrees. Object tube1 has the slightest translation error of 2.02 mm and the slightest orientation error of 11.34 degrees. The object cylinder has the most significant translation and orientation error of 4 mm and 31.87 degrees. The decompositions of the errors are shown in Figure A1.

Despite lacking a standard experiment setup for in-hand object pose estimation, some work has still been explored. The authors of [33] used RGB cameras and GelSights to estimate in-hand object pose with 15 mm accuracy in position and 15 degrees accuracy in orientation. The authors of [34] fused vision and tactile data to obtain 2.99 mm accuracy in position and 8.074 degrees accuracy in orientation. The authors of [21] presented a method based on a bootstrap particle filter to estimate the pose with 2.14−7.31 mm accuracy in position and 0.812−3.392 degrees accuracy in orientation due to the ground truth not detecting almost any rotation. Comparing the state-of-the-art methods, our method obtains a comparable result with a small translation error.

The inner camera can only perceive the objects’ geometric shape and size as the skin isolates the ambient environment. Objects with complex shapes provide abundant shape features and improve the pose estimation accuracy. On the contrary, the geometric shapes of tube4, cylinder, square prism, and triangular prism are more similar to a cylinder, resulting in more significant orientation errors among the seven objects. Those objects are symmetrical, but the features around the symmetry axis lack uniqueness, increasing the difficulty of orientation estimation. The objects’ cross-section shape influences translation error. The columnar objects (tube4, cylinder, and prisms) have a more significant translation error due to their similar cross-section shape among the seven objects.

Objects’ geometric and texture features are essential elements, and the designed finger is limited to obtain the texture as the black coat, influencing the pose estimation precision. A prominent method to improve the precision is to add more features, such as mounting a camera on the gripper or changing the transparent black skin to obtain image features. More features increase the complexity of the device and algorithm but improve performance.

**Figure 10:**
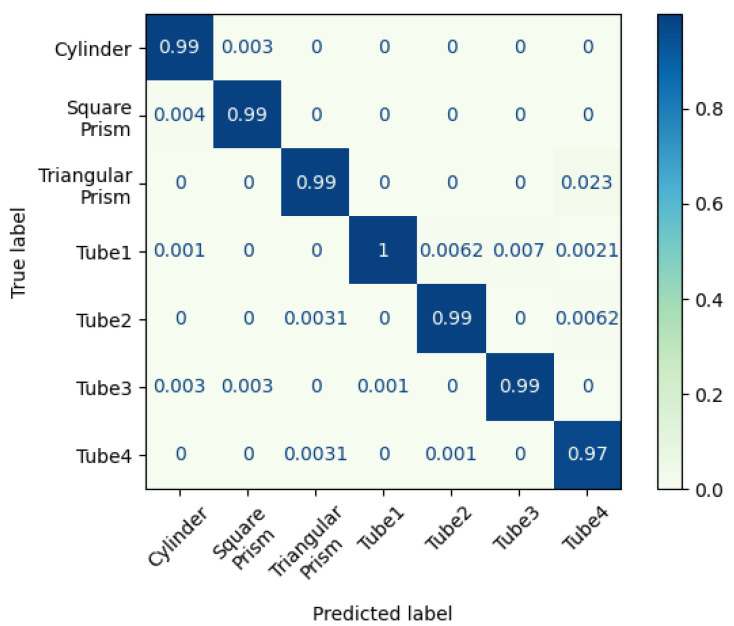
Confusion matrix for object classification.

As the objects have unique 3D shapes and sizes, we obtain a high classification accuracy of 99.05%, as shown in Figure 10. With the proposed method, we can estimate the handled object’s category with a high accuracy proprioceptive touch of the soft fingers.

### 3.3. Reusability and Expansibility of the Framework

As described in the framework, the encoder–decoder architecture reduces the tactile feature dimensions and unites their format for different types of sensors. This makes the tactile information compact and simplifies the processing flow. For other sensors such as GelSight [23], BioTac (https://syntouchinc.com accessed on 19 October 2023) and magnetic skin [35], the different sensing information can also be represented as a latent vector with a convolutional neural network, graph neural network, or other methods according to the data structure.

Then, the extracted tactile features are fused depending on the gripper configuration. In this paper, the fusion features combine two-finger images and gripper configuration and are an input of the auxiliary tasks. For an N-finger gripper, we first extract the tactile information of each finger, then fuse each finger’s features and the gripper configuration, such as joint rotation angles. The gripper configuration represents the joint’s spatial position and can be described as a base pose and the DoF of each finger. As shown in this article, we use AruCo markers to monitor the finger base pose and tactile features of the soft fingers to represent the finger’s DoF, which is independent of hardware.

Finally, the fused features are used for downstream tasks. We demonstrate two basic examples, pose estimation and classification of the handled object, and obtain sound results. We can quickly adapt the frame to other tasks using the same fusion features. Benefitting from the modular design of the framework, we can extract the tactile features independently, fuse them according to the configuration of the hand, and feed them to different auxiliary task models to complete manipulation tasks; this framework applies to scenarios with multi-sensor, multi-gripper, and multi-tasking.

## 4. Conclusions

This paper presents a bio-inspired, soft proprioceptive sensor and a framework for object pose estimation and classification. The proposed soft proprioceptive sensor can be extended to different manipulators, providing extra shape adaptation and interaction information. Based on this sensor, we propose an extendable architecture to extract the tactile information and estimate the handled object’s state. This method achieves a high accuracy of 2.02 mm in translation, 11.34 degrees in orientation, and 99.05% classification accuracy for object classification with an unknown CAD model.

**Figure A2:**
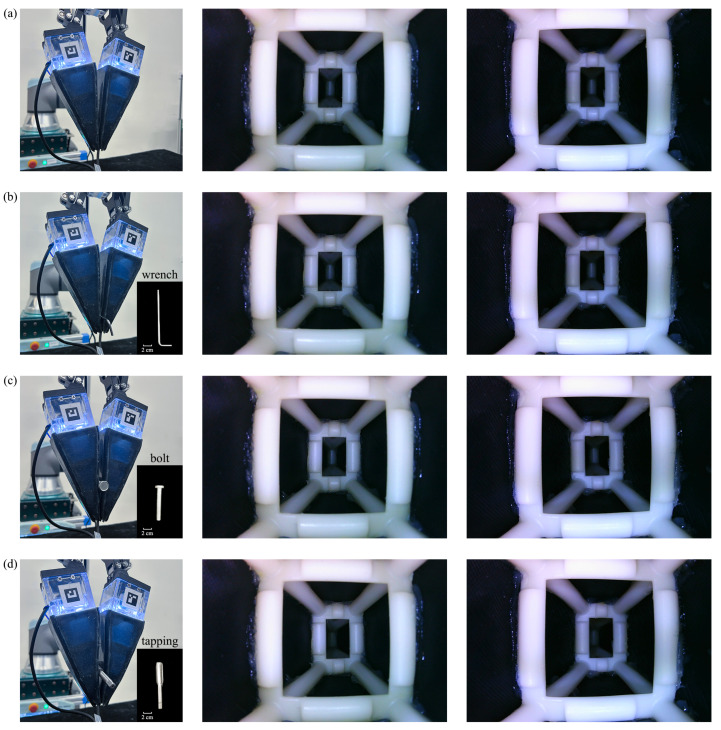
Examples of grasping objects: the left column is an outside image, the middle column is the left inner image, and the right column is the right inner image. (**a**,**b**) are comparisons before and after grasping a small wrench; (**c**) is a comparison grasping a bolt; (**d**) is a comparison grasping a tapping. The deformation of the skeleton is slight after grasping a wrench, and the features of screw threads in (**c**,**d**) are lost in the inner images.

The interaction information is extracted from finger deformations when grasping objects. The pure black skin on the soft finger loses texture features, and the skeleton filters small shape features due to its smooth deformation, as shown in Figure A2. Those properties limit the soft finger to perceiving small objects and distinguishing similar-shaped objects. For unseen objects, our method can not be used directly. Current state-of-the-art pose estimation methods can only handle previously trained objects [36]. Instead of predicting pose directly, [36] proposed a learning-based method that finds corresponding points between an unseen object and an RGBD image, which can be transferred to non-learned objects. However, it depends on the object 3D model and the point cloud of the scene. Our method does not need an object model and uses RGB images only, and it cannot be transferred to non-learned objects easily. For an unseen object, we need to collect data and train the MLP again.

Future work will explore the transferability of this method on different tactile sensors and grippers. This framework provides a uniform feature extractor for different types of tactile sensor information and an extendable structure for different grippers. Meanwhile, more manipulation tasks can be involved with this method.

## Figures and Tables

**Table 1:** Effect of the latent vector dimension.

	Latent Vector Dimension					
	**8**	**16**	**32**	**64**	**128**	**256**
Normalized MSELoss	1.37	1.74	1.36	1.09	1.35	1
Parameters (M)	0.57	0.67	0.88	1.29	2.11	3.74

## Data Availability

Not applicable.
